# The Prospects of Curcumin in Non-Small Cell Lung Cancer Therapeutics

**DOI:** 10.3390/cancers17030438

**Published:** 2025-01-27

**Authors:** Kostas A. Papavassiliou, Amalia A. Sofianidi, Vassiliki A. Gogou, Athanasios G. Papavassiliou

**Affiliations:** 1 First University Department of Respiratory Medicine, ’Sotiria’ Chest Hospital, Medical School, National and Kapodistrian University of Athens, 11527 Athens, Greece; konpapav@med.uoa.gr (K.A.P.); gogvanessa@gmail.com (V.A.G.); 2 Department of Biological Chemistry, Medical School, National and Kapodistrian University of Athens, 11527 Athens, Greece; amsof.00@gmail.com

Lung cancer is the leading cause of cancer-related mortality worldwide, being responsible for approximately 2 million deaths in 2022 [1]. Even though new treatment modalities are constantly being developed, with lung cancer vaccines and antibody-drug conjugates (ADCs) representing the most promising ones [2], overall survival and recovery rates for lung cancer remain significantly low. There is an urgent need for novel and effective therapeutic leads, which will improve the prognosis and quality of life of lung cancer patients. Interestingly, the focus in the therapeutic landscape of many malignancies has recently shifted towards natural compounds and their semisynthetic or synthetic derivatives. Among those, curcumin, a bright yellow polyphenolic compound found in the spice turmeric, has emerged as a promising candidate with diverse biological activities [3]. Curcumin interferes with several cellular signaling pathways involved in cancer development and progression, opening new avenues for the treatment of lung cancer [4]. Herein, we discuss the cellular effects of curcumin and highlight the most recent preclinical evidence on the effectiveness of this natural compound in the treatment of non-small cell lung cancer (NSCLC).

Recent research demonstrates that curcumin is involved in various cellular programmed death processes, including autophagy, apoptosis, pyroptosis, and ferroptosis [5]. More specifically, curcumin can induce autophagy in several cancer models, including NSCLC, resulting in cancer cell death [6]. A recent study also showed that when curcumin activates autophagy, it subsequently triggers the mechanism of ferroptosis in NSCLC cells. Particularly, curcumin possesses the ability to trigger characteristic aberrations associated with ferroptosis in NSCLC cell lines, including the accumulation of oxidative damage, uncontrolled lipid peroxidation, reduction in intracellular glutathione, and mitochondrial dysfunction [7]. Curcumin is also involved in the mechanism of apoptosis by decreasing the levels of 14-3-3 protein, fundamental elements that impede the activation of proapoptotic factors known as BH3-only proteins (Bad) [8]. Notably, curcumin exerts its antitumor activity by modulating apoptosis and autophagy via the phosphatidylinositol 3-kinase (PI3K)/protein kinase B (AKT)/mechanistic target of rapamycin (mTOR) signaling pathway in human lung cancer cell lines [9]. Regarding the process of pyroptosis, curcumin has been found to increase reactive oxygen species (ROS) production, promoting this inflammatory form of cell death in hepatocellular carcinoma (HCC) [10]. What remains to be confirmed is whether these results apply to other malignancies, including NSCLC. Finally, according to a recent study, curcumin has the ability to suppress copper accumulation in NSCLC, which has been shown to affect cancer proliferation and progression [11]. Curcumin binds antioxidant 1 copper chaperone (ATOX1), impeding the ATOX1-mediated copper signaling pathway in NSCLC cells [12].

Epigenetic changes are well established drivers of cancer development and progression [13]. Intriguingly, natural compounds, such as curcumin, have been found to interfere with epigenetic modifications induced in malignant cells, exerting regulatory effects mainly on microRNAs (miRNAs). For instance, a study showed that curcumin inhibits NSCLC cell proliferation, migration, and metastasis by amplifying the expression levels of miR-192-5p. This specific upregulation in turn reduces c-Myc expression and inactivates the Wingless (Wnt)/β-catenin signaling pathway, mitigating NSCLC growth [14]. An additional study showed that curcumin upregulates another miRNA molecule, namely miR-206, which in turn suppresses the activation of the PI3K/AKT/mTOR pathway and, thus, inhibits NSCLC proliferation [15]. Consistent with these findings, researchers demonstrated that curcumin interferes with the Circular RNA hsa_circ_0007580 (circ-PRKCA)/miR-384/integrin subunit beta 1 (ITGB1) pathway [16]. More precisely, curcumin raises the expression of miR-384, decreases circ-PRKCA levels, and downregulates ITGB1 expression; altogether, these modifications lead to NSCLC suppression [16]. Apart from its regulatory effects on miRNA expression, curcumin has also been found to interfere with long non-coding RNAs (lncRNAs). The antitumor effect of curcumin was exerted through augmenting the expression of lncRNA-maternally expressed gene 3 (MEG3) and phosphatase and tensin homolog (PTEN), which sequentially suppressed gemcitabine-resistant NSCLC cell proliferation and activated apoptosis [17].

The aforementioned study is an excellent example of curcumin’s emerging role as a promising agent in overcoming drug resistance of NSCLC to traditional chemotherapy regimens or other novel anticancer agents. Indeed, it was recently demonstrated that curcumin has the ability to abolish the self-renewal potential of the cancer stem cell (CSC) population of NSCLC [18]. This effect was mediated by hindering chemoresistance proteins, aldehyde dehydrogenase, metastasis, angiogenesis, and multiplication of cancer-related proteins, which in turn sensitized NSCLC to cisplatin [18]. A similar study showed that when combining curcumin, cisplatin, and honokiol, another natural compound derived from the traditional Chinese medicine Magnolia, the sensitivity of multidrug-resistant lung adenocarcinoma cells was increased [19]. This in vitro effect was achieved through the inactivation of the AKT/extracellular signal-regulated kinase (ERK) signaling pathway and the regulation of P-glycoprotein, restricting NSCLC progression [19]. Curcumin’s chemosensitizing effects to cisplatin have also been found to be elicited through an endoplasmic reticulum (ER) stress pathway [20]. Further in vitro and in vivo experiments verified the antitumor effects of curcumin in gemcitabine-resistant lung adenocarcinoma cells [21]. The combination of curcumin and gemcitabine proved to be safe and more effective compared to gemcitabine monotherapy in the context of NSCLC [21]. Finally, researchers revealed that curcumin could increase the sensitivity of NSCLC to the tyrosine kinase inhibitor (TKI) crizotinib [22]. The underlying mechanism of this effect lies in curcumin’s ability to silence autophagy by epigenetically modulating the expression of miR-142-5p and its target Unc-51 like autophagy-activating kinase 1 (Ulk1) [22]. Interestingly, curcumin also suppressed the expression of several DNA methyltransferases (DNMTs), including DNMT1, DNMT3A, and DNMT3B, in NSCLC cells, further modifying their epigenetic profile and halting their proliferation [22].

Mounting preclinical evidence suggests the promising antitumor effects of curcumin monotherapy and its combination with established treatment options against NSCLC. However, a huge obstacle in this effort is its poor bioavailability [23], with current efforts oriented towards developing curcumin formulations with better pharmacokinetic profiles. Nanoliposome drug-carrying systems are presently being developed [24] and curcumin’s derivatives are under investigation, offering more satisfactory pharmacokinetic and pharmacodynamic results [25]. Studying the molecular properties of curcumin with the use of artificial intelligence (AI) could trigger the development of better formulations, dosages, and bioavailability formulas and their implementation in clinical practice.
